# The B-cell response to foot-and-mouth-disease virus in cattle following vaccination and live-virus challenge

**DOI:** 10.1099/jgv.0.000517

**Published:** 2016-09

**Authors:** Clare F. J. Grant, B. Veronica Carr, Nagendrakumar B. Singanallur, Jacqueline Morris, Simon Gubbins, Pascal Hudelet, Martin Ilott, Catherine Charreyre, Wilna Vosloo, Bryan Charleston

**Affiliations:** ^1^​ The Pirbright Institute, Ash Road, Woking, UK; ^2^​ CSIRO, 5 Portarlington Road, Geelong, VIC 3220, Australia; ^3^​ Merial Animal Health Ltd, 254 Rue Marcel Mérieux, 69007 Lyon, France; ^4^​ Merial Animal Heath Ltd, Ash Road, Woking, UK

**Keywords:** B cell, FMDV, T-independent, T cell, viral immunology

## Abstract

Antibodies play a pivotal role against viral infection, and maintenance of protection is dependent on plasma and memory B-cells. Understanding antigen-specific B-cell responses in cattle is essential to inform future vaccine design. We have previously defined T-cell-dependent and -independent B-cell responses in cattle, as a prelude to investigating foot-and-mouth-disease-virus (FMDV)-specific B-cell responses. In this study, we have used an FMDV O-serotype vaccination (O_1_-Manisa or O SKR) and live-virus challenge (FMDV O SKR) to investigate the homologous and heterologous B-cell response in cattle following both vaccination and live-virus challenge. The FMDV O-serotype vaccines were able to induce a cross-reactive plasma-cell response, specific for both O_1_-Manisa and O SKR, post-vaccination. Post-FMDV O SKR live-virus challenge, the heterologous O_1_-Manisa vaccination provided cross-protection against O SKR challenge and cross-reactive O SKR-specific plasma cells were induced. However, vaccination and live-virus challenge were not able to induce a detectable FMDV O-serotype-specific memory B-cell response in any of the cattle. The aim of new FMDV vaccines should be to induce memory responses and increased duration of immunity in cattle.

## Introduction

Foot-and-mouth-disease virus (FMDV) is a highly contagious virus infecting cloven-hoofed animals, leading to vesicle formation on the mouth and hooves followed by skin erosions of the cutaneous mucosa. The virus has significant global animal health and socio-economic impact. Maintenance of FMDV-free status is critical for the free trade of animals and animal products (Brehm *et al.*, 2008). One of the main approaches to foot and mouth disease (FMD) control and eradication is through vaccination with inactivated FMDV antigen in formulation with adjuvants (Brehm *et al.*, 2008). However, the current conventional inactivated FMD vaccines only promote relatively short-term humoral immunity with regular repeat vaccinations being required to maintain protective antibody titres (Doel, 1996; Juleff *et al.*, 2009). Serological protection against one FMDV serotype does not confer inter-serotype protection and may not, in some cases, confer intra-serotype protection given the antigenic variation seen within some serotypes (Doel, 1996).

The maintenance of long-term serological protection is provided by plasma cells (antibody-secreting cells, ASCs) and memory B-cells, which act in concert to provide long-term antigen specific antibody production (Ndungu *et al.*, 2009). The nature of the B-cell response is usually dependent upon the requirement of T-cell help to induce a response; these antigens are termed T-dependent (TD) antigens. B-cell responses can also be induced in the absence of T-cell help by type II T-independent (TI-2) antigens, such as viral capsids, that have regular repeating epitopes within their structure. We have previously used model TD and TI-2 antigens to characterize the bovine TD and TI-2 B-cell response, demonstrating that cattle elicit a classical TD B-cell response, but show no detectable TI-2 antigen-specific IgG secreting plasma or memory B-cells post-primary or booster vaccination (Grant *et al.*, 2012).

In cattle, depletion of CD4^+^ T-cells post-vaccination and live-virus challenge has shown that T-cell help is not required to induce protective antibody titres, indicating that the FMDV capsid is a largely TI-2 antigen (Carr *et al.*, 2013; Juleff *et al.*, 2009). Recent studies investigating the early induction of the humoral response to FMDV challenge have demonstrated a rapid induction of localized FMDV-specific plasma cells in secondary lymphoid tissues located in the head and neck of FMDV challenged animals (Pega *et al.*, 2013). However, to date, there is no data on the kinetics of the systemic adaptive immune response in cattle following vaccination and hetero-/homologous live-FMDV challenge. This study has allowed assessment of both the kinetics and magnitude of the FMDV-specific plasma and memory-B-cell responses in peripheral blood post-FMDV vaccination and live-virus challenge in cattle.

## Results

### Clinical outcome following vaccination and live-virus challenge

Following vaccination, the vaccinated animals (FMDV O_1_-Manisa: O1M group; *n*=5 or FMDV O SKR: OSKR group; *n*=5) and the non-vaccinated controls (NVC group; *n*=3) received an intradermolingual live-virus challenge with FMDV-O-SKR virus (OSKRV) at 21 days post-vaccination (21 dpv, 0 days post-challenge, dpc). The animals within the O1M and OSKR groups showed no detectable lesions on the coronary bands and were 100 % protected against live-virus challenge (Table 1). The animals in the NVC group were not protected from live-virus challenge and all three animals showed lesions on all four of their coronary bands, demonstrating dissemination of the FMDV virus (Table 1). All the animals were euthanized on 14 dpc.

**Table 1.** Clinical outcomes post-FMDV O-SKR live-virus challenge Clinical observations of the cattle post-FMDV-O-SKR live-virus challenge. All animals were observed for 8 days post-challenge and the presence and location of lesions were noted for each animal. T = tongue, OSM = oral/snout/mouth, RF = right fore, LF = left fore, RH = right hind, LH = left hind, NVC = non-vaccinated control.

FMDV vaccine	Animal number	Group number	Observation of lesions						Protection status	Protection (%)
			Primary lesions		Secondary lesions					
			T	OSM	RF	LF	RH	LH		
O1-Manisa	FMD182	1	+	–	–	–	–	–	Protected	100
	FMD183		+	+	–	–	–	–	Protected	
	FMD184		+	+	–	–	–	–	Protected	
	FMD185		+	–	–	–	–	–	Protected	
	FMD186		+	–	–	–	–	–	Protected	
O-SKR	FMD197	4	–	–	–	–	–	–	Protected	100
	FMD198		–	–	–	–	–	–	Protected	
	FMD199		+	–	–	–	–	–	Protected	
	FMD200		+	–	–	–	–	–	Protected	
	FMD201		–	–	–	–	–	–	Protected	
NVC	FMD212	7	+	+	+	+	+	+	Unprotected	0
	FMD213		+	+	+	+	+	+	Unprotected	
	FMD214		+	+	+	+	+	+	Unprotected	

### Kinetics of the antigen-specific B-cell response following FMDV O-serotype vaccination and live-virus challenge

The kinetics of the FMDV O_1_-Manisa- and O-SKR-specific IgG plasma (ASCs) and memory B-cell response in peripheral blood was monitored by ELIspot in all three groups following immunization and FMDV-OSKRV live-virus challenge. The results are expressed as the group mean±sd ASCs per 1×10^6 ^PBMCs.

**Fig. 1.**
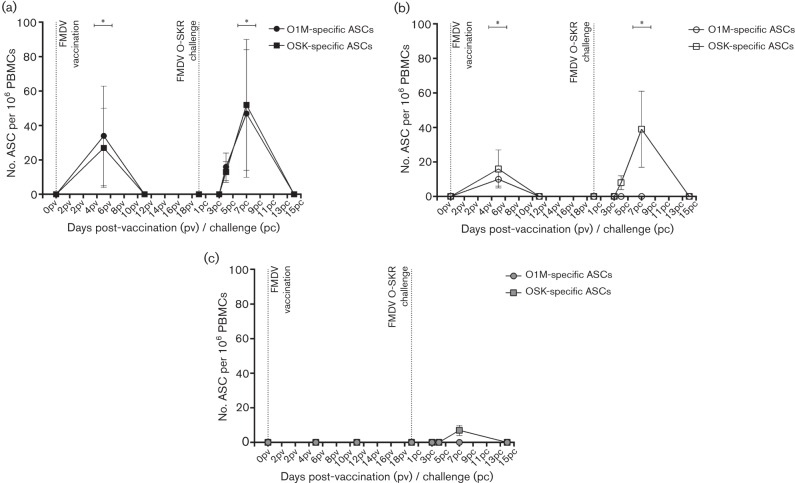
Kinetics of the FMDV-O-serotype-specific plasma-cell response in cattle post-vaccination and live-virus challenge. Kinetics of the O-serotype FMDV-specific plasma-cell response post-vaccination and intradermolingual live-FMDV-O-SKR-virus challenge (indicated by vertical dotted line). (a) FMDV O_1_-Manisa-vaccinated animals (O1M group, black symbols, *n*=5), (b) FMDV O-SKR-vaccinated animals (OSKR group, open symbols, *n*=5) and (c) non-vaccinated controls (NVC group, grey symbols, *n*=3). On all of the graphs, the FMDV O_1_-Manisa-specific ASCs are shown as circle symbols and FMDV-O-SKR-specific ASCs shown as square symbols. Results are expressed as the grouped mean of duplicate determinations from each calf±sd. Statistically significant time points, as compared with 0 dpc (*P*<0.05) are indicated with a * symbol.

Post-vaccination, plasma bursts specific for both O1M and OSKR were observed at 7 dpv in both vaccination groups (Fig. 1a, b, respectively, and Table 2). At 13 dpv, there were no antigen-specific plasma cells detectable in any of the vaccinated animals (Fig. 1, Table 2). There were no detectable FMDV O_1_-Manisa- or O-SKR-specific plasma cells in the NVC group following vaccination (Fig. 1c, Table 2).

At 21 dpv (0 dpc), all of the animals received an intradermolingual live OSKRV challenge. There were no detectable FMDV O_1_-Manisa- or O-SKR-specific plasma cells in any of the three groups at this time point (Fig. 1a, b and c, Table 2). Following challenge, a burst of both FMDV O_1_-Manisa- and O-SKR-specific plasma cells was observed between 4 dpc and 7 dpc, which peaked at −7 dpc in the O1M group (O1M: 47±37, OSKR: 52±38 -specific ASCs per 10^6^ PBMCs, *P*<0.05, Fig. 1a, Table 2). By 14 dpc, there were no antigen-specific plasma cells detectable in the peripheral blood of these animals.

In the OSKR group following challenge, a plasma-cell burst specific for FMDV O-SKR only was observed from 3 dpc to 7 dpc, again, peaking at 7 dpc (OSKR: 39±22 -specific ASCs per 10^6^ PBMCs, *P*<0.05, Fig. 1b, Table 2), and by 14 dpc, there were no detectable FMDV O-SKR-specific plasma cells. No FMDV O_1_-Manisa-specific plasma cells were detected after OSKRV challenge in the OSKR group.

The NVC group also showed a burst of only FMDV- O-SKR-specific plasma cells at 7 dpc, at a much lower level than the vaccinated groups (NVC group: OSKR: 7±3 -specific ASCs per 10^6^ PBMCs, Fig. 1c, Table 2), which was undetectable by 14 dpc (Fig. 1c, Table 2).

The presence of antigen-specific memory B-cells was monitored in all animals following both vaccination and FMDV-OSKRV challenge. However, no FMDV O_1_-Manisa or O-SKR-specific memory B-cells were detected in any of the vaccinated or non-vaccinated groups at any time point following either vaccination or live-virus challenge. There was also no increase in the total number of IgG-secreting memory B-cells in any of the groups following vaccination or live-virus challenge.

In a previous pilot study, a detectable memory-B-cell response was present in the peripheral blood of cattle at 7 days post-booster vaccination (#16 : 7 and #18 : 34 FMDV O_1_-Manisa specific ASCs per 10^6^ cultured PBMCs). The memory-B-cell response was still detectable at 14 days post-booster vaccination in one animal (#16 :  4 FMDV O1-Manisa specific ASCs per 10^6^ cultured PBMCs).

**Table 2.** Total number of IgG FMDV-O-serotype ASCs in PBMC population Total number of IgG FMDV O_1_-Manisa and O-SKR-specific ASCs post-vaccination and live-virus challenge. Results are expressed as the grouped mean of duplicate determinations from each animal±sd.

Days post-vaccination (Days post-live-virus challenge)	Total number of FMDV-O-serotype-specific ASCs per 10^6^ PBMC					
	O1M group (*n*=5)		OSKR group (*n*=5)		NVC group (*n*=3)	
	O1M	O-SKR	O1M	O-SKR	O1M	O-SKR
0	0	0	0	0	0	0
7	34±29	27±23	10±4	16±11	0	0
13	0	0	0	0	0	0
21 (0pc)	0	0	0	0	0	0
24 (3pc)	0	0	0	0	0	0
25 (4pc)	16±8	13±6	0	8±4	0	0
28 (7pc)	47±37	52±38	0	39±22	0	7±3
35 (14pc)	0	0	0	0	0	0

### Kinetics of the serological response following FMDV-O-serotype vaccination and live-virus challenge

The serological responses to FMDV O_1_-Manisa and O-SKR were also monitored following vaccination and FMDV O-SKR live-virus challenge using the liquid-phase-blocking ELISA (LPBE) and virus-neutralization test (VNT).

#### LPBE-based serology.

The LPBE was used to quantitatively assess the titre of antibodies present in the serum that are capable of binding to either the FMDV O_1_-Manisa or O-SKR virus. An LPBE titre of greater than 1 in 40 (expressed as a log_10_ titre of 1.60) is associated with a positive response to vaccination (Kitching *et al.*, 2008). The grouped results are expressed as the log_10_ titre group average±sd.

**Fig. 2.**
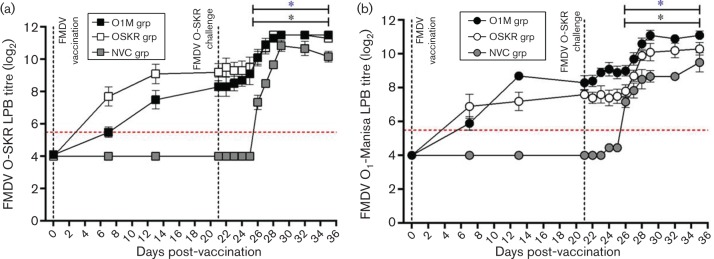
Kinetics of the FMDV O-serotype-specific liquid-phase-blocking antibody response in cattle post-vaccination and live-virus challenge. Kinetics of the (a) FMDV O-SKR-specific and (b) FMDV O_1_-Manisa-specific liquid-phase-blocking antibody titres post-vaccination and FMDV O-SKR live-virus challenge in the FMDV O_1_-Manisa vaccinated cohort (black symbols), the FMDV-O-SKR-vaccinated cohort (open symbols) and the non-vaccinated controls (grey symbols). Results are expressed as the log_10_ transformed grouped mean of duplicate determinations from each calf±sd Statistically significant time points, as compared with 0 dpc (*P*≤0.05), indicated by * symbol (FMDV O_1_-Manisa vaccinated cohort = black, FMDV O-SKR vaccinated cohort = blue).

The O1M and O-SKR vaccinated groups developed blocking antibodies that were specific for both FMDV O-SKR and O_1_-Manisa following vaccination (Fig. 2a, b, respectively). The kinetics of the response was similar in both of the vaccinated groups following vaccination and challenge, with an increase in both FMDV O_1_-Manisa- and O-SKR-specific LPBE titres from 7 dpv (O1M group: 1.77±0.27 and 1.65±0.22 and O-SKR group: 2.08±0.48 and 2.32±0.39 against FMDV O_1_-Manisa and FMDV O-SKR, respectively), with a further increase by 13 dpv (O1M group: 2.62±0.13 and 2.26±0.38 and O-SKR group: 2.17±0.36 and 2.74±0.40 against FMDV O_1_-Manisa and FMDV O-SKR, respectively). On the day of OSKRV challenge, the titres were similar between the vaccinated groups (21 dpv/0 dpc; O1M group: 2.50±0.27 and 2.50±0.39 and O-SKR group: 2.29±0.16 and 2.77±0.36 against FMDV O_1_-Manisa and FMDV O-SKR, respectively). Following OSKRV challenge, there was an increase in blocking antibody titres for both vaccinated groups from 6 dpc, which didn’t change significantly until the end of the study at 14 dpc (Fig. 2).

The NVC group had no detectable antibodies until 5 days after OSKRV challenge, when antibodies specific for both O_1_-Manisa and FMDV O-SKR (NVC group: 2.16±0.18 and 2.21±0.23 against FMDV O_1_-Manisa and FMDV O-SKR, respectively) were found by LPBE. The titres continued to increase against O_1_-Manisa until 14 dpc (NVC group: 2.86±0.30, Fig. 2a), whereas against FMDV-O-SKR titres peaked at 8 dpc (NVC group: 3.26±0.17, Fig. 2b), and then decreased until the end of the study at 14 dpc (NVC group: 3.06±0.17, Fig. 2b). There was a significant difference between the LPBE FMDV-OSKR titres observed in the vaccinated and the NVC groups at 5 to –7 dpc (*P*<0.05, Fig. 2b).

#### VNT-based serology.

**Fig. 3.**
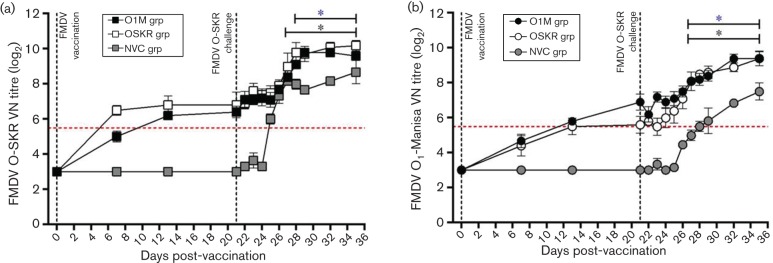
Kinetics of the FMDV-O-serotype-specific virus-neutralizing antibody response in cattle post-vaccination and live-virus challenge. Kinetics of the O-serotype (a) FMDV O-SKR-specific and (b) FMDV O_1_-Manisa-specific virus neutralization (VN) titre post-vaccination and FMDV-O-SKR live-virus challenge in the FMDV O_1_-Manisa-vaccinated cohort (black symbols), the FMDV O-SKR-vaccinated cohort (open symbols) and the non-vaccinated controls (grey symbols). Results are expressed as the log_10_ transformed grouped mean of duplicate determinations from each calf±sd. Statistically significant time points, as compared with 0 dpc (*P*≤0.05), indicated by * symbol, (FMDV O_1_-Manisa vaccinated cohort = black, FMDV O-SKR-vaccinated cohort = blue).

The VNT was used to quantitatively assess neutralizing antibody titres towards FMDV O_1_-Manisa or O-SKR *in vitro* and was expressed as the log_10_ titre group average±sd with titres greater than 1 in 32 (expressed as a titre of 1.5 1) considered as a positive response to vaccination (positive threshold applied at The Pirbright Institute)(Martin & Chapman, 1961). Following vaccination and OSKRV challenge, the kinetics of both the FMDV O_1_-Manisa- and O-SKR virus neutralizing (VN) titres were similar in both vaccinated groups of animals, demonstrating an increase in both FMDV O_1_-Manisa- and O-SKR-specific titres from 7 dpv (Fig. 3). Although there was an increase in neutralizing antibodies observed by 7 dpv, the titres were only considered positive across both vaccinated groups by 13 dpv (O1M group: 1.74±0.08 and 1.87±0.17; OSKR group: 1.65±0.32 and 2.05±0.35 VN titres against FMDV O_1_-Manisa and FMDV O-SKR, respectively), with the exception of vaccine-homologous VN titres in the OSKR group, which were positive by 7 dpv (1.96±0.19 FMDV-O-SKR VN titres; Fig. 3b). The titres for O_1_-Manisa and FMDV O-SKR remained elevated until the OSKRV challenge at 21 dpv (O1M group: 2.08±0.31 and 1.92±0.22; O-SKR group: 1.68±0.33 and 2.05±0.49 against FMDV O_1_-Manisa and FMDV O-SKR, respectively). Similar to the blocking antibodies, an increase in neutralizing antibodies was observed in both vaccinated groups from 5–14 dpc. There were no significant differences between the end-point VN titres obtained in either of the vaccine groups (O1M group: 2.82±0.27 and 2.88±0.13; O-SKR group: 2.83±0.33 and 3.06±0.20 against FMDV O_1_-Manisa and FMDV O-SKR, respectively).

The NVC group developed neutralizing antibodies specific for O_1_-Manisa by 7 dpc (1.65±0.15 FMDV O_1_-Manisa VN titres), but significantly lower compared with the same time point in both of the vaccinated groups (*P*<0.05, Fig. 3a). The O-SKR VN titres in the NVC animals increased from 4 dpc (1.80±0.15 FMDV O-SKR VN titres) and remained elevated until 14 dpc, reaching similar end-point titres as the vaccinated groups. However, the FMDV-O-SKR VN titres were significantly lower in the NVC group at 7, 8 and 11 dpc as compared with the same time points in the vaccinated animals (*P*<0.05, Fig. 3b).

### Correlation between the serological response to FMD and antigen-specific plasma-cell number

When comparing the plasma-cell burst and end-point antibody titres following vaccination, there was no significant correlation (*P*=0.11) between the number of plasma cells at the peak of the primary burst (7 dpv, Fig. 1a, b) and the end-point neutralizing titre (14 dpc, Fig. 3). In contrast, there was a positive correlation between the 7 dpv plasma-cell burst and the LPBE titre at 14 dpc (rho-0.41, *P*<0.05, Fig. 2). After live-virus challenge, there was no significant correlation between the post-challenge peak of plasma-cell burst (4 and 7 dpc) and the neutralizing end-point titres (14 dpc). However, there was a significant positive correlation between the plasma-cell number at both 4 dpc (rho=0.70, *P*<0.001) and 6 dpc (rho=0.74, *P*<0.001), with the LPBE end-point titre (14 dpc).

## Discussion

This is the first study to assess the kinetics and magnitude of the FMDV-specific plasma and memory-B-cell response following FMD vaccination and a homologous and heterologous live FMDV challenge.

Following immunization with either FMDV O-SKR or O_1_-Manisa vaccines, there was an expansion of FMDV O_1_-Manisa- and O-SKR-specific plasma cells at 7 dpv, which was coupled with an increase in both the VN and blocking-antibody titres specific for both FMDV O-serotypes. The timing of the antigen-specific plasma-cell burst following primary immunization is in keeping with previously published data in both cattle (Grant *et al.*, 2012; Juleff *et al.*, 2009) and humans (Bernasconi *et al.*, 2002; Halliley *et al.*, 2010; Kelly *et al.*, 2006; Wrammert *et al.*, 2008). The presence of both FMDV O-SKR-specific ASCs and antibodies following FMDV O_1_-Manisa vaccination indicates that the FMDV O_1_-Manisa vaccine is able to induce a protective cross-reactive plasma-cell response specific for FMDV O-SKR. Similarly, the FMDV O-SKR vaccinated group had a burst of FMDV O_1_-Manisa specific ASCs, again indicating that the inactivated FMDV O-SKR vaccine was also able to induce a cross-reactive plasma-cell response.

At 21 days after vaccination, all of the animals were challenged with live FMD-O-SKR virus. This resulted in a detectable antigen-specific plasma-cell burst between 4 and 7 dpc, which is consistent with previously published data demonstrating an increase in IgG FMDV-specific ASCs in bovine lymphoid tissues from 4 to 6 dpc (Pega *et al.*, 2013). However, the antigen specificity of the plasma-cell burst post-live-virus challenge differed between the vaccinated groups. The FMDV O_1_-Manisa vaccinated animals developed a burst of ASCs specific for both FMDV O_1_-Manisa and O-SKR that was coupled with an increase in both blocking and virus-neutralizing antibodies specific for both FMDV O-serotypes tested, whereas, the OSKR group demonstrated only an FMDV O-SKR-specific plasma-cell burst post-challenge, despite the induction of FMDV O_1_-Manisa-specific plasma cells post-vaccination. However, there was an increase in both the blocking and neutralizing antibodies specific for FMDV O_1_-Manisa following live-virus challenge, despite the lack of detectable FMDV O_1_-Manisa-specific plasma cells. This indicates that there is a compartment of ASCs that was not detectable in the peripheral blood.

The non-vaccinated control animals showed FMDV-O-SKR-specific plasma-cell burst at 7 dpc, which was delayed compared with the vaccinated cohorts. The timing of the plasma-cell burst in these animals is consistent with a naïve B-cell response to the FMDV-O-SKR-virus challenge and is in keeping with previously published data in cattle (Grant *et al.*, 2012; Juleff *et al.*, 2009) and humans (Lee *et al.*, 2010). The magnitude of the FMDV-O-SKR-specific plasma-cell burst at this time point was lower compared with the same time point in the vaccinated cohorts, which is surprising considering the equivalent antibody response post-infection. The non-vaccinated animals showed an increase in both virus-neutralizing and blocking-antibody titres against FMDV O-SKR, reaching titres that were comparable with the vaccinated animals. These infected animals also developed FMDV O_1_-Manisa-specific blocking and neutralizing antibodies despite the lack of detectable O_1_-Manisa-specific plasma cells.

It has previously been shown that following FMDV challenge, there is a rapid induction of FMDV-specific ASCs in lymphoid tissues draining the infection site, where FMDV undergoes proliferation (Pega *et al.*, 2013), which are likely to be short-lived extra-follicular plasma cells which remain at the site of induction (McHeyzer-Williams, 2003). Taken together, our data suggest that there are ASCs that are not detected in the peripheral blood, which are likely to be within the local lymphoid tissue draining at the site of infection (Pega *et al.*, 2013, 2015).

The positive correlation between the magnitude of the plasma-cell burst and the total amount of antibody capable of binding the virion (blocking-antibody titre) is in keeping with previously published vaccination data in cattle and humans (Juleff *et al.*, 2009; Kelly *et al.*, 2006; Nieminen *et al.*, 1998). However, the lack of correlation between the plasma-cell response and the virus-neutralizing antibody titre again indicates that the number of antigen-specific B-cells detected in the periphery does not correlate with the total pool of antibody-secreting plasma cells, also shown in humans (Baumgarth, 2013).

T-independent type 2 (TI-2) antigens have regularly spaced repeating epitopes that are able to stimulate B-cells in the absence of CD4^+^ T helper cells (Feldmann & Easten, 1971). The FMDV capsid has structural features that lend it towards stimulating B-cells in a TI-2 manner. It has previously been noted that acute cytopathic viral infections result in the accelerated induction of antibody in a T-independent manner (Fehr *et al.*, 1996; Lee *et al.*, 2005), providing a rapid means of stopping systemic spread of the virus (Bachmann & Zinkernagel, 1997). In the absence of CD4^+^ T-cells, cattle can produce class-switched antibody rapidly in response to FMDV challenge, thus demonstrating the largely T-independent nature of FMDV (Juleff *et al.*, 2009). Pega and colleagues have also demonstrated that rapid induction of FMDV-specific plasma cells occurs in local lymphoid tissue following live-virus challenge, which, again, is consistent with a T-independent response (Pega *et al.*, 2013). The current study adds further evidence to support the hypothesis that FMDV is a largely TI-2 antigen, as there were no detectable FMDV-specific memory B-cells in any of the cattle, following either vaccination or live-virus challenge. Our previous work has shown that immunization of cattle with a model TI-2 antigen also resulted in an undetectable antigen-specific memory B-cell response (Grant *et al.*, 2012). These findings are also consistent with other TI-2 antigen vaccination regimes in humans (Kelly *et al.*, 2006). Recent work by Pega and colleagues also showed a lack of memory B-cells specific for FMDV following vaccination in cattle (Pega *et al.*, 2015). However, a small number of FMDV-specific memory B-cells were detected in the peripheral blood of cattle that had received multiple FMDV vaccinations (Pega *et al.*, 2015). We have also confirmed this finding, showing a small number of FMDV-specific memory B-cells circulating in cattle that have received two FMDV vaccinations. Thus, it has been hypothesized that the sustained antibody response seen in cattle that have recovered from FMDV infection (Cunliffe, 1964), is the result of continuous stimulation of short-lived plasma cells generated at the site of antigen persistence (and viral replication) (Juleff *et al.*, 2008). It is also possible that the low (or undetectable) number of a antigen-specific memory-B-cell response is due to memory B-cells that have not entered the peripheral blood. Indeed, Aiba and colleagues have demonstrated that antigen-specific memory B-cells generated following immunization preferentially localized to a survival niche adjacent to contracted germinal centres in mouse spleens, rather than entering the circulating memory-B-cell pool (Aiba *et al.*, 2010). This has not been ruled out in this study, as only the peripheral blood of the cattle was tested.

To conclude, this study has demonstrated that both the FMDV O_1_-Manisa and O-SKR vaccines are able to provide anamnestic response during FMDV challenge. The data from this study also showed that the current inactivated FMDV vaccine and, indeed, live-virus challenge are unable to induce a detectable memory-B-cell response in the peripheral blood. Thus, to further improve the FMDV vaccine to increase the duration of immunity, the selected antigen should seek to induce long-lived plasma and memory B-cells, signatures of a TD response.

## Methods

### Virus, vaccines, antigens and cells.

The vaccines used in this study were double-oil emulsion monovalent O_1_-Manisa/Turkey/69 (>6 PD_50_) and O/SKR/2010 (>6 PD_50_), supplied by Merial Animal Health, Pirbright, UK. The challenge FMDV (species *Foot-and-mouth-disease virus*, genus *Aphthovirus*, family *Picornaviridae*, order *Picornavirales*) was cattle derived (FMDV O/SKR/8/11), provided by Merial Animal Health Ltd, Pirbright, UK. For the cellular assays (ELIspot and Proliferation assays), inactivated antigen from each vaccine strain was provided by Merial Animal Health Ltd, (O_1_ Manisa/Turkey/69 at 450 µg ml^−1^ and O/SKR/2010 at 17.4 µg ml^−1^). For the virus-neutralization assays, IBRS-2 cells were used with O_1_ Manisa/Turkey/69 and O/SKR/8/11 FMDV strains.

### Calves, vaccination and live-virus challenge protocol.

Thirteen 6-month-old conventionally reared Holstein-Friesian male calves (*Bos taurus*, The Pirbright Institute, Woking, UK) were split into two groups of five and a control group of three animals. Each of the vaccine groups were immunized intramuscularly with a full dose of either inactivated FMDV O_1_-Manisa vaccine (O1M group) or inactivated FMDV O-SKR vaccine (OSKR group). The controls were not vaccinated (NVC group).

All calves (O1M, OSKR and NVC groups) were challenged intradermolingually with 0.2 ml live-FMDV O-SKR virus (OSKRV) into two different sites on the upper surface of the tongue (0.1 ml per site, challenge dose of 10^5.7^ ID50) at 21 days post-vaccination (dpv), (0 days post-challenge, dpc).

All animals were observed and the general health status, hoof sensitivity and presence of tongue and hoof lesions were recorded for 8 dpc. Dissemination of infection from the site of inoculation was determined by looking for the presence of lesions on the coronary bands, which indicated a lack of protection.

Heparinized peripheral blood and serum samples were taken from all animals at 0, 7, and 13 dpv, daily from 0–8 dpc, and at 10 and 14 dpc.

To validate the detection of FMDV-specific memory B-cells using the ELIspot assay, two additional 6-month-old conventionally reared Holstein-Friesian male calves (#16 and #18, *Bos taurus*, The Pirbright Institute) were prime and booster vaccinated intramuscularly with 10 µg FMDV capsid material (Porta *et al.*, 2013) in Montinide ISA 201 VG adjuvant (Sepic, Paris, France). The vaccines were administered intramuscularly 21 days apart and peripheral blood was taken at regular intervals following both primary and booster vaccinations.

All experiments were approved by the Pirbright Institute’s and the CSIRO-Australian Animal Heath’s ethical review processes and were in accordance with national guidelines on animal use.

### FMDV O_1_-Manisa- and O-SKR-specific ELIspot for the detection of plasma or cultured antibody secreting cells (ASCs).

The ELIspot assay is utilized in this study to provide a method of enumerating FMDV-specific cells that are actively secreting antibody. Plasma cells spontaneously secrete antibody and so can be detected by B-cell ELIspot in a freshly isolated PBMC population. By contrast, memory B-cells are quiescent and require stimulation to induce differentiation into ASCs prior to detection using the B-cell ELIspot assay. This differentiation is performed using a 6-day culture condition which provides an antigen-independent polyclonal stimulation to promote memory B-cell differentiation into spontaneously antibody-secreting plasma cells (Lefevre *et al.*, 2009). The 6-day culture period is also long enough to ensure the depletion of pre-exisitng ASCs that would have been present in the PBMC population (Lefevre *et al.*, 2009).

Bovine PBMCs were isolated from heparinized blood samples (Grant *et al.*, 2012). The FMDV-specific ELIspot was performed using both freshly isolated and 6-day stimulated PBMCs for the detection of FMDV-specific plasma and memory B-cells, respectively. The validation and details of the 6-day stimulated PBMC culture conditions for the detection of memory B-cells by ELIspot are detailed in Lefevre and colleagues (Lefevre *et al.*, 2009) and the FMDV-specific ELIspot assay was adapted from the protocol detailed by Grant and colleagues (Grant *et al.*, 2012). Briefly, the ELIspot plates were set up using MultiScreenHA plates (Millipore, Watford, UK) coated with 100 µl per well of a ^1^/_5000_ dilution of rabbit anti-FMDV O1 UKG polyclonal antibody (Pirbright Institute) in 0.1 M carbonate buffer (pH 9.6) for 2 h at 37 ^o^C. Plates were washed five times in PBS and then blocked using 4 % dried milk (Marvel, Premier Foods, St Albans, UK) in PBS, followed by five more washes in PBS. Inactivated FMDV antigens (FMDV O_1_-Manisa or O-SKR, Merial Animal Health Ltd) were added to the plate (100 µl per well of 1 µg ml^−1^ diluted antigen in PBS). The plates were washed and stored at 4 ^o^C until required. A total IgG control ELIspot was also performed for both the freshly isolated and 6-day stimulated PBMCs (Grant *et al.*, 2012).

The PBMCs were suspended at 5×10^6^ cells ml^−1^ and 1 : 2 serial dilutions were performed in ELIspot medium down to 1.5×10^5^ cells ml^−1^. Incubation and the ‘spot’ detection steps of the ELIspot were performed as previously described by Grant *et al*. (2012). During the live-virus challenge phase of the study, after washing in tap water, the ELIspot plates were submerged in 4 % paraformaldehyde (PFA, Sigma-Aldrich) for 1 h, prior to air drying overnight.

### Foot-and-mouth disease virus (FMDV) O_1_-Manisa- and O-SKR-specific virus-neutralization test (VNT) and liquid-phase-blocking ELISA (LPBE).

All serological assessments (VNT and LPBE) were performed at the FMDV World Reference Laboratory (FMDWRL, The Pirbright Institute).

The VNT and LPBE assays were performed using the same stock viruses as the vaccine antigens provided by Merial Animal Health Ltd. The virus-neutralizing activity of the sera was determined using the VNT assay methodology described in the OIE *Manual of Diagnostic Tests and Vaccines for Terrestrial Animals* (Kitching *et al.*, 2008) and the end-point titres were calculated according to the method of Reed and Muench (Reed & Muench, 1938). The LPBE was performed according to the methodology described by Hamblin and colleagues (Hamblin *et al.*, 1986). All results were expressed as the grouped mean titre±standard deviation (sd).

### Statistical analysis.

To establish if there was a correlation between the FMDV-specific antibody titres and the peak number of plasma cells generated following both vaccination and live-virus challenge, a Spearman's rank correlation coefficient (rho) was computed between the size of the plasma cell burst at the peak of the burst post-vaccination (7 dpv) and post-live-virus challenge (25 dpv and 28 dpv) versus both the end-point LPBE and VNTs titres (35 dpv). Statistically significant increases in ASC number, LPBE and VN titres and PBMC proliferation were calculated by comparing 0 dpc with the time point of interest using a one-way ANOVA followed by Dunnett’s multiple comparison test using Graphpad Prism software (GraphPad Prism version 6.00 for Windows, GraphPad Software, La Jolla, California USA).
